# Scaling Approaches for Pediatric Dose Selection: The Fremanezumab (AJOVY^®^) Journey to Select a Phase 3 Dose Using Pharmacokinetic Data from a Phase 1 Study

**DOI:** 10.3390/pharmaceutics13060785

**Published:** 2021-05-24

**Authors:** Aksana Jones, Orit Cohen-Barak, Andrijana Radivojevic, Jill Fiedler-Kelly

**Affiliations:** 1 Cognigen Corporation, Buffalo, NY 14221, USA; jill.fiedler-kelly@cognigencorp.com; 2 Teva Pharmaceutical Industries, Netanya 4250483, Israel; Orit.Cohen-Barak@teva.co.il; 3 IntiGrowth LLC, New York, NY 10025, USA; Andrijana.Radivojevic@intigrowth.com

**Keywords:** pediatric dose selection, fremanezumab, pharmacometrics, pediatric migraine

## Abstract

Fremanezumab, a fully humanized IgG2Δa/kappa monoclonal antibody, selectively targets the calcitonin-gene-related peptide (CGRP) and prevents it from binding to the CGRP receptor. The safety, tolerability, pharmacokinetics (PK), and efficacy of fremanezumab for treating migraines administered as a once monthly 225 mg dose or a once quarterly 675 mg dose have been well characterized in adults. The fremanezumab exposure and body weight relationship supported the use of the approved 225 mg monthly adult dose for pediatric patients weighing ≥45 kg. In the pediatric Phase 3 program, a 120 mg dose for patients weighing <45 kg was determined using the results of an open-label study and a population PK modeling and simulation strategy. A thorough evaluation was conducted to further characterize the population PK of fremanezumab and assess the predictive performance of the adult population PK model when applied to the Phase 1 pediatric data, the predictive performance of alternative pediatric population PK models, and the predictive performance of the selected pediatric population PK model via a noncompartmental-based approach. This latter comparison to noncompartmental results provided additional evidence that the pediatric population PK model predicts the observed data well and supports the 120 mg monthly dose in patients weighing <45 kg.

## 1. Introduction

Migraine is a condition characterized by attacks of headache and associated symptoms (such as nausea, photophobia, or phonophobia). Among populations of children of all ages, migraine prevalence ranges from 8% to 11% [1,2,3,4,5]. The prevalence of migraine is substantially lower among children younger than 7 years of age, ranging from 1% to 3% [6]. The prevalence of migraine in children younger than 12 years of age is less than 1/3 of the prevalence among adolescents [4,5,7,8]. Therefore, the prevalence of migraine increases throughout childhood, with estimates for adolescents comparable to the 12% to 15% prevalence estimates cited for adult populations [5,9,10,11].

Calcitonin gene-related protein (CGRP) is a well-studied neuropeptide that plays an important role in the etiology of migraines, both centrally and peripherally [12,13]. Jugular levels of CGRP are increased during migraine attacks, and intravenous (iv) CGRP administration induces migraine-like headaches in most individuals with migraine [14,15]. Inhibition of CGRP pathways has demonstrated efficacy in the treatment of episodic [16,17,18,19] and chronic [20] migraine.

Fremanezumab is a fully humanized IgG2Δa/kappa monoclonal antibody (mAb) derived from a murine precursor. Fremanezumab selectively targets CGRP and has been approved by the United States Food and Drug Administration for the preventive treatment of migraine in adults [21,22]. Fremanezumab binds to the CGRP ligand and blocks both the α- and β-CGRP isoforms from binding to the CGRP receptor [21]. Introduction of two mutations into the constant region of the fremanezumab heavy chain limits antibody effector functions, thereby preventing fremanezumab from stimulating antibody-dependent cell-mediated cytotoxicity and triggering complement-mediated lysis [23].

During the development of fremanezumab, pharmacometric modeling and simulation has been used extensively to support development-related decision-making. A population pharmacokinetic (PK) model was iteratively developed, initially using available data from healthy adult subjects in two Phase 1 studies following iv and subcutaneous (sc) administration [24] and adult migraine patients with episodic migraine (EM) and chronic migraine (CM) after sc administration in two Phase 2b studies [19,20,25], and was refined with data from three Phase 3 trials in adults [26,27]. Inclusion of Phase 1 data after iv and sc administration supported estimation of bioavailability. The final population PK model [28] provided a basis for prediction of subject-specific exposure estimates to support assessment of exposure-response relationships for efficacy and safety endpoints.

A 75 mg dose was selected for a pediatric Phase 1 study using a simulation strategy based on the previously developed adult population PK model. However, results from the Phase 1 study which aimed to characterize the PK, safety, and tolerability of sc administration of fremanezumab in pediatric migraine patients (6 to 11 years of age, inclusive) indicated that a fremanezumab dose of 75 mg sc monthly in pediatric patients weighing <45 kg may not be adequate [29].

The previously developed adult fremanezumab population PK model was refined based on the sparsely sampled concentration data from the pediatric Phase 1 study in order to support a dosing recommendation for pediatric patients weighing <45 kg in the Phase 3 program [29]. While the updated population PK model was found to adequately fit the pediatric data, a thorough assessment was conducted to evaluate additional modeling assumptions. After assessing the predictive performance of the previously developed adult population PK model applied to the pediatric data, a thorough assessment of pediatric dose selection of fremanezumab was then performed by considering revisions to the initially developed pediatric population PK model [29]. The additional revisions to the initially developed pediatric population PK model included (1) the use of the conventional theoretical allometric exponents (0.75 for clearance [CL]; 1.0 for central volume of distribution [V_c_]), (2) assuming specified fixed adult values (i.e., fixed bioavailability [F1] and absorption lag time [ALAG1]) based on the previously developed adult population PK model, and (3) the use of less informative prior information (i.e., 25% RSE [relative standard error expressed as a percent]) to allow more weight to the data collected from the pediatric Phase 1 study. The current analysis describes the thorough assessment conducted to further support the selection of an appropriate dose(s) for Phase 3 development of fremanezumab in pediatric patients with migraine.

## 2. Materials and Methods

### 2.1. Overview of the Pharmacokinetic Model Development Process

The Phase 1 study that provided the pediatric pharmacokinetic data used to re-estimate and refine the adult population PK model has previously been reported in detail [29]. Briefly, the Phase 1 study included patients aged 6 to 11 years old who were diagnosed with migraine (International Classification of Headache Disorders, ICHD-3 [30]) and weighed at least 17.0 kg and less than 45.0 kg. Patients with any prior exposure to a mAb targeting the CGRP pathway were not permitted. As part of the inclusion criteria, patients needed to be in good health as determined by a medical and psychiatric history, physical examination, 12-lead electrocardiogram, and clinical laboratory tests including serum chemistry, hematology, coagulation, and urinalysis. Written informed consent was obtained from each patient’s parent(s)/legal representative(s) and each pediatric patient (if the patient was able) before any study procedures or assessments were done. The participants were divided into two weight groups of male and female patients, 17 to <30 kg (*n =* 6) and ≥30 to <45 kg (*n =* 9). Each patient received a single 75 mg dose of fremanezumab administered as an sc injection into the abdomen. Pharmacokinetic sampling comprised five samples per patient, on Days 2, 11, 29, 85, and 113 post-dose. Data utilized in the creation of the PK analysis dataset included dosing information (amount and time), PK sampling information (time relative to last dose and concentration of fremanezumab), and demographic data. All patients with at least one measurable fremanezumab concentration were included. Individual patients’ body weight, age, and sex were evaluated as potential predictors of variability in the PK model parameters. All covariates were documented at baseline and assumed to have remained constant throughout the study period. Fremanezumab concentration data consisted of 75 samples collected from all 15 patients enrolled in the Phase 1 pediatric study. A validated chemiluminescence enzyme-linked immunosorbent assay (ELISA) was used for the determination of fremanezumab plasma concentrations. The lower limit of quantitation value for the PK assay was 0.25 μg/mL [31].

### 2.2. Pediatric Population Pharmacokinetic Model Development

All exploratory data analyses and presentations of data were performed using SAS Version 9.4 (SAS Institute, Cary, NC, USA) and KIWI Version 4 (Cognigen Corporation, Buffalo, NY, USA) [32,33]. Population modeling was performed using the computer program NONMEM, Version 7, Level 3.0 (ICON Development Solutions LLC, Hanover, MD, USA) [34]. NONMEM analyses were performed on an Intel cluster with the Linux operating system.

The first assessment of the pediatric population PK model development began with a model applying the previously developed adult population PK model [28] to the data from patients who participated in the pediatric Phase 1 study [29]. The previously developed adult population PK model, including estimates of PK parameters (typical value estimates) and between-subject variability in PK parameters, was used to describe the PK data collected in the pediatric Phase 1 study without performing an estimation step (MAXEVAL = 0 option in NONMEM).

In a following step, the fremanezumab adult population PK model [28] was used as prior information for fitting the pediatric PK data. The NONMEM $PRIOR subroutine, which is a restricted maximum likelihood function for constraining parameter estimates based on prior knowledge, was used to implement the informative priors for the population mean PK parameters. The first-order conditional estimation with interaction (FOCEI) method was used during all stages of the model development process.

The base model was a two-compartment model with first-order absorption and elimination. Log-normally distributed interindividual variability (IIV) for CL, V_c_, and first-order absorption rate constant (k_a_) were included. Covariates already included in the previously developed model (allometric weight scaling on CL and V_c_) were re-evaluated for precision and plausibility of parameter estimates based on the pediatric data. Initial residual variability (RV) was described with an additive plus proportional model.

Following refinement of the population PK model based on the pediatric data from the Phase 1 study, a step-wise forward selection (α = 0.01 plus at least a 5% reduction in IIV in the parameter of interest) followed by backward elimination (α = 0.001) methodology was used for the re-assessment of body weight, age, and sex as covariate effects on PK model parameters.

### 2.3. Evaluation of Different Revisions to the Pediatric Population Pharmacokinetic Model

After assessing the predictive performance of the previously developed adult population PK model applied to the pediatric data, a thorough assessment of pediatric dose selection of fremanezumab was then performed by considering revisions to the initially developed pediatric population PK model [29].

The following revisions of the pediatric population PK model were evaluated:

- Fixed allometric exponents: applying conventional theoretical allometric exponents (0.75 for CL; 1.0 for V_c_);
- Fixing the F1 and ALAG1 parameters to the estimates obtained in adults;
- Using less informative adult priors (25% RSE).

### 2.4. Model Evaluation

The adequacy of the population PK models was evaluated using a simulation-based, prediction-corrected visual predictive check (pcVPC) method [35,36]. Monte-Carlo simulations were performed by simulating 1000 datasets identical in structure to the original dataset using NONMEM. Statistics of interest were calculated from the simulated and observed data for comparison, e.g., the 10th, 50th (median), and 90th percentiles of the distributions of simulated and observed concentrations were compared. These percentiles were plotted versus time, with the original observed dataset and/or percentiles based on the observed data overlaid to visually assess concordance between the model-based simulated data and the observed data. Model evaluation was performed throughout the modeling steps via pcVPC, which provided a graphical model performance assessment.

Following the thorough assessment, the PK exposure measures derived based on the developed pediatric population PK model were compared to the observed PK exposure measures derived by noncompartmental analysis (NCA) to assess the predictive performance of the developed pediatric population PK model. Based on a virtual pediatric population, simulated exposure measures (i.e., area under the concentration-time curve from time 0 to 28 days (AUC_28d_) and maximum drug concentration (C_max_)) were calculated after a single dose for the virtual pediatric patients and compared to observed exposure measures derived by NCA in the pediatric population after receiving a single 75 mg dose of fremanezumab and using a similar PK sampling scheme as in the pediatric Phase 1 PK study.

### 2.5. Simulations

A virtual population of 2400 pediatric male and female patients (6 to 17 years of age) was generated (200 patients per year of age) and used along with the pediatric PK model estimates from each alternative model to simulate concentration-time data for monthly sc doses ranging from 60 to 225 mg [29]. Body weight was determined by the Centers for Disease Control and Prevention (CDC) growth chart [37] using a random number to determine the Z score (percentile of body weight) for each simulated age according to their corresponding sex. Simulated exposure measures (i.e., average concentration (C_av_), cumulative exposure represented by the AUC_28d_, minimum drug concentration (C_min_), and C_max_) were calculated at steady state for the virtual pediatric patients and compared to exposure measured at steady state in the adult population receiving fremanezumab 225 mg sc monthly.

## 3. Results

### 3.1. Previously Developed Adult Model Applied to Pediatric Data

**Figure 1:**
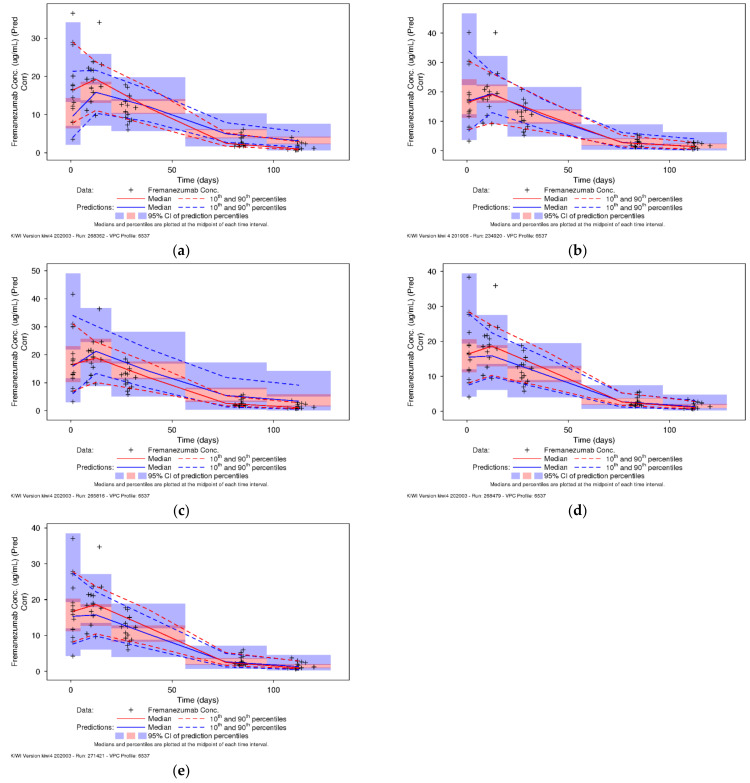
Prediction-corrected visual predictive check plots for pediatric patients (*n =* 15): (**a**) applying the previously developed adult population pharmacokinetic model, (**b**) final fremanezumab pediatric population pharmacokinetic model ^1^, (**c**) alternative fremanezumab pediatric population pharmacokinetic model with fixed allometric exponents, (**d**) revised fremanezumab pediatric population pharmacokinetic model with bioavailability and lag time fixed to adult values, and (**e**) revised fremanezumab pediatric population pharmacokinetic model with fixed bioavailability and lag time from adults and less informative priors (25% relative standard error [RSE]). CI: confidence interval, Conc: concentration, Pred Corr: prediction-corrected. ^1^ Subfigure b was reproduced with permission from [29], SAGE Publications, 2021.

The ability of the previously developed adult population PK model to describe fremanezumab plasma concentrations measured in pediatric patients who participated in the Phase 1 study [29] was evaluated by applying the model [28] to the data with no estimation step (MAXEVAL = 0). Goodness of fit (GOF) for the previously developed population PK model applied to the data from the pediatric patients with all parameters fixed was evaluated graphically (data on file Cognigen Corp.) and suggested that there is an apparent underprediction for the higher fremanezumab concentrations observed in the pediatric patients. This underprediction indicates that the previously developed adult population PK model is not able to describe data outside of the previously observed baseline body weight range (43.5 to 131.8 kg) in adults well. Simulations were then performed (1000 replicates) using the previously developed adult population PK model applied to the data from the pediatric patients (*n =* 15), and the pcVPCs for fremanezumab plasma concentrations versus time were plotted and visually inspected (Figure 1a). A clear underestimation of C_max_ is apparent with this model, as well as the apparent inability to describe the central tendency and overall variability of the observed data in pediatric patients. This confirmed the necessity for model refinement of the adult population PK model based on pediatric data.

### 3.2. Pediatric Population Pharmacokinetic Model Development

The previously published pediatric population PK model, developed with the inclusion of PK data from the Phase 1 pediatric study, was found to provide an adequate fit to the pediatric data, supporting its use for simulations and to recommend doses for the pediatric Phase 3 fremanezumab studies [29].

**Figure 2:**
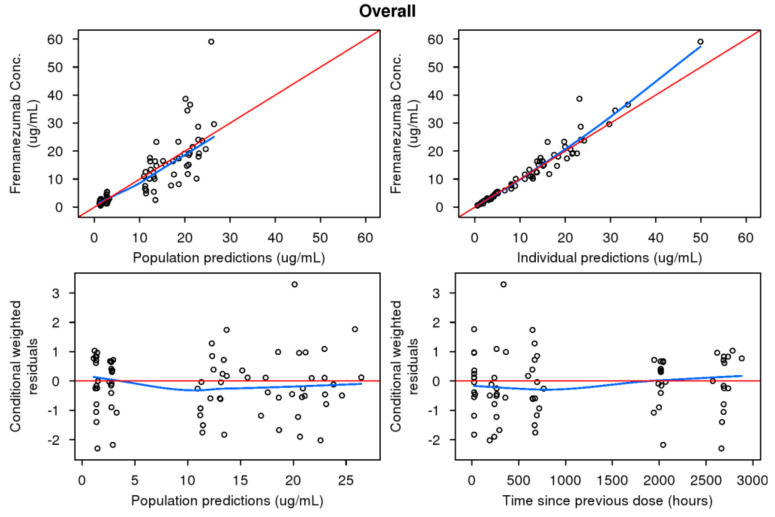
Goodness-of-fit diagnostic plots for the fremanezumab pediatric population pharmacokinetic model. Conc: concentration.

Table 1 provides the parameter estimates for the final pediatric model used to recommend doses for the pediatric Phase 3 fremanezumab studies: a two-compartment model with proportional residual variability [29]. Parameters for intercompartmental clearance (Q) and peripheral volume of distribution (V_p_) were fixed to those estimated based on the previously developed adult PK model [28]. All fixed effect parameters were estimated reasonably well with ≤32.7% RSE; random effect parameters were estimated with less precision (ranging from 43.7% RSE for RV to 53.2% RSE for IIV on CL). However, despite a low condition number, indicative of a lack of over-parameterization, high correlations (|*r*| > 0.9) between CL and V_c_ were consistently observed, indicating limitations in the informativeness of the sparse pediatric data relative to all PK parameters. Bayesian shrinkage was very low (i.e., 0.13% for CL and 7.3% for V_c_). Higher variability was noted during the absorption phase in pediatrics, when compared to adults, but, with the limited data available, was not able to be estimated reliably. The magnitude of unexplained variability in PK parameters ranged from 79.9 %CV (coefficient of variation expressed as a percent) (V_c_) to 34.2 %CV (CL). Epsilon shrinkage was low (<19%), and RV was estimated to be 18.4 %CV across the fremanezumab concentration levels. The GOF plots for the final pediatric base PK model (Figure 2) indicate that the PK model provided a reasonable fit to the data. Following covariate analysis, only an additional effect of age on V_c_ was statistically significant, but, due to the high correlation between age and body weight (|*r*| = 0.89) in this pediatric population and the inclusion of the effect of weight on V_c_, the age effect was deemed not clinically relevant and was not retained in the model [29].

The pcVPC method was performed using the final pediatric population PK model to ensure adequate model performance and to assess the predictive capabilities of the model. The median and 90% prediction interval, derived from the simulated datasets, overlaid on the observed fremanezumab concentration data in pediatric patients and corresponding percentiles, are provided in Figure 1b, which was previously published [29]. In general, the pcVPC results indicate that the model adequately predicts both the central tendency of the concentration data over time as well as the extent of variability in the observed pediatric data.

### 3.3. Evaluation of Different Revisions to the Pediatric Population Pharmacokinetic Model

The estimated PK parameter values and their associated variabilities were compared between the adult model, the pediatric model (with allometric exponents estimated), and each of the alternative models explored as shown in Table 1.

#### 3.3.1. Revised Pediatric Model with Fixed Allometric Exponents

An alternative pediatric population PK model using allometric exponents fixed to the conventional theoretical values (0.75 for CL; 1.0 for volume) was used to re-estimate the pediatric data. Comparing the final parameter estimates from the alternative model with the allometric exponents fixed to the literature values, it is shown that the estimates for CL and V_c_ (i.e., the parameters representative of the typical values at the median body weight (71 kg)) were quite similar, which could be attributed to the use of the informative prior information for the population mean PK parameters from the previous adult population PK model despite fixing the allometric exponents to 0.75 for CL and to 1 for V_c_. However, it should be noted that the IIV in CL increased by 19.6 %CV with the model with fixed exponents (i.e., from 34.2 %CV with the model using non-fixed exponents to 53.8 %CV using the fixed exponents). Based on standard criteria comparing the value of the objective function (VOF) for the model with fixed allometric exponents (two degrees of freedom) to the model with estimated exponents, the model using fixed allometric exponents would not be selected as preferential (i.e., VOF increase of 13.386 points, *p* = 0.00124). Visual inspection of the pcVPC for the model with fixed allometric exponents (Figure 1c) shows that the model with fixed allometric exponents overpredicted the central tendency of the observed data in the 15 pediatric patients 6 to 11 years of age weighing <45 kg.

#### 3.3.2. Revised Pediatric Model with Bioavailability and Lag Time Fixed to Adult Values

In the previously developed adult population PK model [28], the F1 of fremanezumab after sc administration in healthy subjects (following 225 and 900 mg) was estimated at 65.8% (based on population PK analysis). Applying the adult population PK model to the observed pediatric patient data clearly showed an underestimation of C_max_ (Figure 1a) and, hence, indicated a potential difference in absorption in the pediatrics; however, the assumption of 65.8% F1 of fremanezumab and a fixed lag time of 0.0803 days was applied and the revised model was assessed for its predictive performance. When comparing the final parameter estimates from the pediatric model with fixed F1 and lag time to the estimates from the adult model applied to the pediatric population (Table 1), it is apparent that the estimates for CL and V_c_ (i.e., the parameters representative of the typical values at the median body weight (71 kg)) are very close, while the allometric exponents are quite different (i.e., 0.642 versus 1.05 for the CL allometric exponent and 2.56 versus 1.53 for the V_c_ allometric exponent, respectively). These differences in the allometric exponents again indicate that the relationships between body weight and CL and V_c_ are different in patients weighing <45 kg, as observed in the pediatric Phase 1 study, when compared to the previously extrapolated body weight relationships based on adult data, where the minimum body weight was 43.5 kg. Visual inspection of the pcVPC for the model with fixed F1 and lag time (Figure 1d) indicates that the model generally underpredicts the observed data in the 15 pediatric patients 6 to 11 years of age weighing <45 kg, especially in the early part of the profile (i.e., during absorption). This underprediction further confirms the necessity to remove the lag time in the pediatric population PK model, as well as supports the assumption that the F1 in pediatric patients might be higher when compared to adults and, hence, the pediatric F1 should be treated as apparent (F1 = 1). Furthermore, it was shown previously that no absorption lag time was observed in some of the pediatric patient data, meaning some patients’ profiles exhibited an apparent C_max_ by Day 2, possibly also due to the limited PK sampling and the first measurable sample post-dose being on Day 2.

#### 3.3.3. Pediatric Model with Fixed Bioavailability and Lag Time from Adults and Less Informative Priors (25% RSE)

Lastly, a model allowing less informative prior information from the adults on fixed effect parameters (25% RSE) was explored due to the highly informative priors from the adult model (where %RSEs ranged from 1.5% to 12.2% [28]). The objective of using less informative priors was to give relatively more weight to the data collected in pediatric patients participating in the Phase 1 study. Using 25% RSE for priors on all fixed effect parameters led to slightly different PK parameter estimates (Table 1) when compared to the other alternative models investigated. However, poor precision of IIV on V_c_ (138% RSE) still indicates that there is little information to allow precise estimation of all the IIV terms. Previously, multiple attempts were made to estimate the IIV in k_a_ in the pediatric population, but, likely due to the limitations of available PK concentrations during the absorption phase (only one PK sample at Day 2), it was determined to be non-identifiable in the pediatric data. Since both k_a_ and V_c_ are PK parameters associated with characterization of the absorption phase of a drug and due to the limitations of available PK concentrations during the absorption phase in the current pediatric study, it is anticipated that either IIV on V_c_ or IIV on k_a_ will not be identifiable. Visual inspection of the pcVPC for the model with fixed F1 and lag time and less informative priors (Figure 1e) indicates that the model still underpredicts the observed data in the 15 pediatric patients 6 to 11 years of age weighing <45 kg, despite the less informative priors and corresponding changes to the PK parameter estimates. This leads to the conclusion that despite the various attempts to utilize the prior information from the adult population PK model, some adjustments in the assumptions would need to be made to allow precise and robust estimation in the pediatric population PK model and provide a better description of the central tendency and overall variability observed in the pediatric patients.

### 3.4. Predictive Performance of Pediatric Model to Support Phase 3 Development

As an additional assessment to the model evaluations via pcVPC, the model-based exposure measures after a single 75 mg dose were simulated for the pediatric patients from the Phase 1 study, as well as for the virtual pediatric patients weighing <45 kg and compared to the NCA results from the Phase 1 study to further confirm model appropriateness. For these simulations, a sparse sampling schedule (Days 2, 11, 29, 85, and 113 postdose) consistent with that used in the pediatric Phase 1 study was applied to the simulated data. Table 2 shows the comparison of the observed C_max_ and AUC_28d_ based on the NCA, as well as simulated C_max_ and AUC_28d_ based on the pediatric population PK model. It is shown that the simulated PK exposures based on the pediatric population PK model using a sparse PK sampling scheme are comparable to the observed PK exposures derived by the NCA while using the patients from the pediatric Phase 1 study as well as virtual pediatric patients. In addition to the previously outlined model evaluations, this further confirms the very good predictive performance of the pediatric population PK model with respect to PK exposure measures, C_max_ and AUC_28d_, and supports the corresponding dose selection based on this model.

### 3.5. Simulation-Based Dose Selection for Pediatric Patients Weighing <45 kg

As previously reported [29], a virtual population of 2400 pediatric patients (aged 6 to 17 years) was generated (200 patients per year of age) and used along with the final pediatric PK model estimates to simulate concentration-time data for the virtual pediatric patients following administration of monthly sc doses ranging from 60 to 225 mg. For these simulations, weight was determined by the CDC growth chart [37] using a random number to determine the Z score (percentile of body weight) for each simulated age.

Exposure measures, derived from the simulated concentration data, were then calculated in the virtual pediatric patients and compared to exposures achieved at the 225 mg sc monthly dose in the adult population. Among the 2400 virtual pediatric patients 6 to 17 years of age, 1453 had a body weight that was <45 kg. For pediatric virtual patients weighing <45 kg (*n =* 1453), the median (range) baseline body weight was 29.0 (17.0 to 44.8) kg for patients 6 to 17 years of age.

For pediatric patients 6 to 17 years of age with baseline weight <45 kg administered 120 mg sc monthly, the simulated AUC_28d_ distribution was nearly identical to the adult patient distribution following administration of 225 mg sc monthly [29]. Although the simulated C_max_ distribution following 120 mg sc monthly in the pediatric population suggests slightly higher C_max_ than that achieved in the adult population following 225 mg sc monthly, overall, the upper exposure range extended only slightly above the upper range of the adult exposures. Similar results were observed for the other PK exposure parameters (C_av_, C_min_, and C_max_). Based on the similarity in exposures over the body weight range spanning from 17 to 45 kg, no additional body weight cutoff value was deemed necessary.

Despite the apparent mis-specifications of the different revisions to the pediatric population PK model, the model with fixed allometric exponents and the model with bioavailability and lag time fixed to adult values were carried forward to simulate fremanezumab concentrations for each virtual pediatric patient following administration of sc doses ranging from 60 to 225 mg monthly to enable comparison of predicted doses for pediatric patients weighing <45 kg.

**Figure 3:**
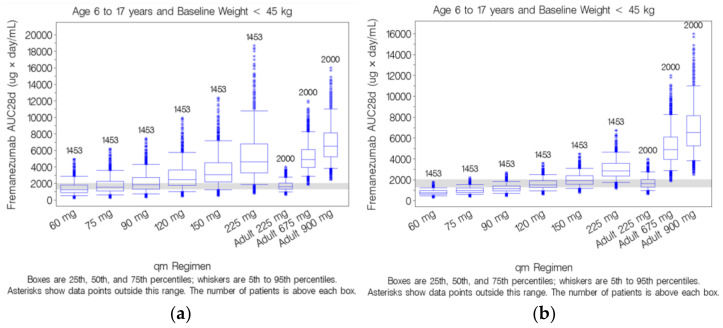
Simulated steady state fremanezumab AUC_28d_ for pediatric patients 6 to 17 years of age with baseline body weight <45 kg compared to the adult distribution following fremanezumab 225 mg subcutaneous monthly administration based on different revisions to the pediatric population pharmacokinetic model: (**a**) fixed allometric exponents ^1^, (**b**) bioavailability and lag time fixed to adult values ^1^. AUC_28d_: area under the concentration time curve from time 0 to 28 days, qm: monthly. ^1^ Subfigures a and b were reproduced with permission from [29], SAGE Publications, 2021.

Boxplots of simulated steady-state fremanezumab AUC_28d_ by dose for pediatric patients 6 to 17 years of age with a baseline body weight of <45 kg based on the model with fixed allometric exponents were compared to the adult AUC_28d_ distributions following 225 mg sc monthly, as shown in Figure 3a. Based on the model assuming fixed allometric exponents of 0.75 for CL and 1 for V_c_, administration of 75 mg fremanezumab sc monthly in pediatric patients 6 to 17 years of age with a baseline body weight of <45 kg is predicted to achieve a similar distribution of simulated AUC_28d_ to the adult patient distribution following administration of 225 mg sc monthly. However, considering the apparent overprediction of exposures with this model based on the pcVPC plots (Figure 1c), we believe that the overprediction of AUC_28d_ and C_max_ leads to an underprediction of the corresponding dose needed to achieve exposures comparable to those in adults receiving 225 mg sc fremanezumab, in effect supporting a dose higher than 75 mg in pediatric patients weighing <45 kg. Furthermore, with the fixed allometric exponent model, the consequence of a pediatric patient reaching a body weight of 45 kg would entail a three-fold increase in monthly sc fremanezumab dose.

Boxplots of simulated steady-state fremanezumab AUC_28d_ by dose for pediatric patients 6 to 17 years of age with a baseline body weight of <45 kg based on the model with F1 and ALAG1 fixed to adult values, compared to the adult AUC_28d_ distributions following 225 mg sc monthly, are shown in Figure 3b. Despite the apparent underestimation (Figure 1d), for pediatric patients 6 to 17 years of age with a baseline body weight of <45 kg administered 120 mg sc monthly, the distribution of simulated AUC_28d_ is nearly identical to the adult patient distribution following administration of 225 mg sc monthly. These results confirm that the 120 mg dose selection would still hold true for the pediatric model using a fixed F1 of 0.658 and a fixed ALAG1 of 0.0803 days based on the previously developed adult population PK model with corresponding estimated allometric exponents of 0.642 and 2.56 for CL and V_c_, respectively, based on the pediatric data.

## 4. Discussion

Prior to the execution of the pediatric Phase 1 study, pediatric doses were selected using a modeling and simulation approach based on the adult population PK model [28]. Given the anticipated similarity between adult and pediatric patients with migraine and the effect of body weight on fremanezumab PK in adults, exposure measures were simulated in a virtual pediatric population by extrapolating to the appropriate pediatric body weight values. Using exposure matching to the efficacious adult dose of 225 mg sc monthly, a dose of 75 mg sc monthly in pediatric patients between 6 and 11 years weighing <45 kg was selected for implementation in the pediatric Phase 1 study [29].

Upon completion of the Phase 1 study, the goal of the initial analysis was to update and refine the previously developed adult population PK model for fremanezumab using observed data collected from pediatric patients with migraine in this study. An apparent difference in the absorption was observed in the pediatric patients from the pediatric Phase 1 PK study when compared to the adult fremanezumab concentration-time profiles. However, the adult population PK model [28] was used in this analysis as informative prior information for estimating the population mean PK parameters in the pediatric PK data [29].

The ability of the adult population PK model to describe the PK data in pediatric patients was assessed in the context of the current analysis. This assessment was essentially based on pcVPC plots. The pcVPCs indicated that the adult PK model was not able to fully describe the central tendency and overall variability observed in the pediatric PK data and further confirmed the necessity of updating the population PK model based on the pediatric data. Re-estimation of the model using informative priors from the adult model led to a more accurate description of the pediatric PK data, with parameter estimates still close to the previous estimates with the exception of the allometric exponents for CL and V_c_. Both the allometric exponents for body weight on CL and V_c_ decreased when compared to those estimated from the adult model. Body weight ranged from 21.4 to 44.9 kg in the 15 pediatric patients, thus informing the parameter estimates of the allometric exponents to better predict over this range of weight distribution. Model refinement utilizing the prior information based on the adult population PK model was further explored. Notably, fixing F1 and ALAG1 based on the adult population PK model was assessed and did not lead to significant model improvement. Finally, allowing less informative prior information (25% RSE) on fixed effect parameters was also explored since previous priors from the adult population PK model [28] were highly informative (with %RSEs ranging from 1.5% to 12.2%). This resulted in different estimates for CL, V_c_, and k_a_, as well as allometric exponents, when compared to previous models. However, poor precision of IIV on V_c_ (138 %RSE) still indicated that there was little information to allow precise estimation. Furthermore, the model still exhibited underprediction of the pediatric data (Figure 1e), despite less informative priors and changes to the estimates. More importantly, reducing the weight of prior information was associated with changes in PK parameter estimates, specifically the allometric exponents. Overall, it was shown consistently across all explored models, including data from the 15 patients who participated in the pediatric Phase 1 study, that the relationship of body weight as a significant predictor of variability in PK, with respect to both CL and V_c_, differs from that observed in the adult population and confirms that the relationship should be adjusted based on the observed pediatric PK data. This is consistent with the general clinical pharmacology considerations for pediatric study guidelines [38], to inform the population PK model based on emerging data to aid in optimizing pediatric dosing strategies and concurrently using PK data from the pediatric studies to confirm PK estimates in the various age and corresponding body weight subgroups.

In comparison to model-based predictions based on either the adult PK model alone or the application of theoretical assumptions regarding scaling into a pediatric population, we demonstrated that utilization of the pediatric data to inform the estimation of the allometric exponents is warranted (based on the pcVPCs) and provides a prediction that is more reflective of the anticipated relationship of CL and V_c_ with pediatric body weight and, hence, translates into more realistic exposures in pediatric patients weighing <45 kg. In addition, several mAbs have utilized allometric scaling approaches in the prediction of pediatric doses with estimated exponents ranging from −0.313 to 0.882 for CL and ranging from −0.233 to 1.22 for V_c_, indicating that the conventional theoretical allometric exponents of 0.75 for CL and 1.0 for V_c_ may not always be reflective of the true relationship between body weight and the corresponding PK parameter [39,40,41].

The use of population PK modeling, combining prior knowledge from adults with available, albeit sparse and limited, data from pediatric patients, is a frequently implemented approach described in United States Food and Drug Administration reviews and labels for mAbs evaluated in pediatric populations [42]. Furthermore, population PK approaches are encouraged and widely accepted for the estimation of PK parameters in pediatrics [38]. Despite the small number of pediatric subjects included in the Phase 1 study, the use of these valuable data, informative of observed PK characteristics in these pediatric subjects, should not be minimized. The Bayesian approach used to estimate fremanezumab PK in pediatric subjects was selected in an attempt to balance the prior knowledge in adults and the informational content of the limited observed pediatric data.

Given the comparison of the models with estimated and fixed allometric exponents and in consideration of the wide safety margin for fremanezumab with considerable evidence supporting the safety of the approved dose of 225 mg sc monthly in adults, the expected exposures in pediatric patients weighing <45 kg after administration of 120 mg sc monthly fall well within the exposure range of adults receiving doses up to 900 mg sc monthly, regardless of the allometric scaling approach used. Therefore, while taking into account that the 120 mg sc monthly dose in pediatric patients weighing <45 kg is predicted to result in median exposures at least 1.8-fold lower than the adult 675 mg sc monthly regimen and to minimize the risk of decreased efficacy in this pediatric population, the 120 mg sc monthly dose is recommended for pediatric patients weighing <45 kg. Furthermore, the model confirms the very good predictive performance of the pediatric population PK model with respect to a comparison of model-based PK exposure measures (calculated based on the sparse-sampling strategy, as implemented in the Phase 1 pediatric study) and the NCA-based estimates from this study. This further supports the dose selection of 120 mg for pediatric patients 6 to 17 years of age weighing <45 kg based on the pediatric population PK model with estimated allometric exponents.

While the proposed dose of 120 mg sc monthly for pediatric patients differs from the initial proposed dose of 75 mg sc monthly for pediatric patients 6 to 17 years of age and weighing <45 kg, this change can be justified by the inclusion of the pediatric data into the population PK model. The results from the current analysis indicate that the pediatric population PK model provided a reasonable fit to the pediatric data. Alternative models, based on differing assumptions, all failed to result in an improved fit to the pediatric data. Furthermore, both the central tendency of the concentration data over time, as well as the extent of variability in the observed pediatric data, were adequately predicted, supporting the use of the pediatric population PK model for simulations and dose selection decision-making. Overall, these analyses supported the selection of fremanezumab 120 mg sc monthly in pediatric patients weighing <45 kg that is being evaluated in two global Phase 3 studies (NCT04458857, NCT04464707).

## 5. Conclusions

A thorough assessment of pediatric dose selection of fremanezumab was performed by considering revisions to the initially developed pediatric population PK model. Alternative models, based on differing assumptions, all failed to result in an improved fit to the pediatric data. Furthermore, both the central tendency of the concentration data over time, as well as the extent of variability in the observed pediatric data, were adequately predicted, supporting the use of the initial pediatric population PK model for simulations and dose selection decision-making.

The NCA-based approach provides additional evidence that the pediatric population PK model is able to predict the observed data well and supports the dose selection of 120 mg monthly in patients weighing <45 kg, which matches the desired exposures from the approved adult 225 mg sc monthly dose. Overall, it was shown that the final pediatric population PK model is robust in describing the observed pediatric data from subjects who participated in the pediatric Phase 1 study. The data obtained in the pediatric Phase 3 program will further enrich the pediatric population PK model, and model refinement may be warranted based on emerging data.

## Figures and Tables

**Table 1:** Overall comparison of parameter estimates and standard errors for fremanezumab comparing the previously developed adult population pharmacokinetic model with the various approaches for the pediatric population pharmacokinetic model.

Parameter	Previously Developed Adult Model Applied to Pediatric Data	Pediatric Model to Support Phase 3 Development ^1^	Revised Pediatric Model with Fixed Allometric Exponents	Revised Pediatric Model with Bioavailability and Lag Time Fixed to Adult Values	Pediatric Model with Fixed Bioavailability and Lag Time from Adults and Less Informative Priors (25% RSE)
CL: central clearance (L/day)	0.0902 (1.50)	0.0905 (0.0937)	0.0907 (0.213)	0.0905 (0.305)	0.106 (13.1)
CL: allometric exponent for weight (−)	1.05 (4.33)	0.245 (32.7)	0.750 (FIXED)	0.642 (10.6)	0.833 (16.9)
Vc: central volume of distribution (L)	1.88 (3.38)	1.89 (0.213)	1.90 (0.569)	1.89 (0.512)	2.18 (4.57)
Vc: allometric exponent for weight (−)	1.53 (10.3)	1.20 (32.7)	1.00 (FIXED)	2.56 (8.03)	2.70 (6.66)
k_a_: absorption rate constant (1/day)	0.180 (12.2)	0.252 (14.8)	0.262 (10.5)	0.176 (2.72)	0.178 (5.40)
Q: intercompartmental clearance (L/day)	0.262 (FIXED)	0.262 (FIXED)	0.262 (FIXED)	0.262 (FIXED)	0.262 (FIXED)
V_p_: peripheral volume of distribution (L)	1.72 (FIXED)	1.72 (FIXED)	1.72 (FIXED)	1.72 (FIXED)	1.72 (FIXED)
F1: bioavailability	0.658 (FIXED)	Apparent (F1 = 1)	Apparent (F1 = 1)	0.658 (FIXED)	0.658 (FIXED)
ALAG1: lag time (day)	0.0803 (FIXED)	Removed	NE	0.0803 (FIXED)	0.0803 (FIXED)
	Magnitude of Interindividual Variability (%RSE)				
Interindividual variability in CL	23.4 %CV (4.60)	34.2 %CV (53.2)	53.8 %CV (31.2)	24.9 %CV (68.3)	23.6 %CV (63.8)
Interindividual variability in V_c_	35.1 %CV (19.9)	79.9 %CV (49.6)	80.8 %CV (51.4)	58.9 %CV (133)	56.1 %CV (138)
Interindividual variability in k_a_	59.0 %CV (15.8)	NE	NE	28.4 %CV (57.5)	27.9 %CV (59.5)
	Parameter Estimate (%RSE)				
Residual variability proportional component	0.0531 (4.03)	0.0338 (43.7)	0.0333 (43.8)	0.0795 (69.5)	0.0793 (68.9)
Residual variability additive component	0.204 (25.6)	NE	NE	NE	NE
VOF	NA	201.396	214.782	219.479	218.022

%CV: coefficient of variation expressed as a percent, NA: not applicable, NE: not estimated, %RSE: relative standard error expressed as a percent, VOF: value of objective function. ^1^ The results presented in this column for the pediatric model to support Phase 3 development were previously published [29]. Note: the gray shaded rows highlight the allometric exponents used in the various approaches for the pediatric population pharmacokinetic model.

**Table 2:** Noncompartmental sparse sampling comparison, based on observed and simulated fremanezumab concentrations in pediatric patients receiving a single 75 mg dose of fremanezumab.

Body Weight	Pharmacokinetic Parameter	Pediatrics		
		<30 kg	>30 kg	All Patients
Number of Patients		6	9	15
NCA (observed) [29]	C_max_ (µg/mL) mean (SD)	34.2 (13.5)	16.3 (5.27)	23.5 (12.8)
	AUC_28d_ (µg × h/mL) mean (SD)	16,200 (5310)	8840 (3080)	11,800 (5410)
NCA (predicted based on pediatric population pharmacokinetic model)	C_max_ (μg/mL) mean (SD)	31.8 (9.91)	17.9 (5.02)	23.5 (9.93)
	AUC_28d_ (μg × h/mL) mean (SD)	14,200 (2120)	9620 (3070)	11,500 (3520)
Number of Simulated Patients		786	667	1453
Simulation NCA (based on pediatric population pharmacokinetic model)	C_max_ (µg/mL) mean (SD)	28.4 (9.11)	22.6 (7.06)	25.7 (8.73)
	AUC_28d_ (µg × h/mL) mean (SD)	13,200 (2880)	11,200 (2730)	12,300 (3000)

AUC_28d_: area under the concentration-time curve from time 0 to 28 days, C_max_: maximum drug concentration, NCA: noncompartmental analysis, SD: standard deviation.

## Data Availability

Qualified researchers may request access to patient-level data and related study documents, including the study protocol and statistical analysis plan. Requests will be reviewed for scientific merit, product approval status, and conflicts of interest. Patient-level data will be de-identified and study documents will be redacted to protect the privacy of trial participants and to protect commercially confidential information. Please email USMedInfo@tevapharm.com to make your request.
